# An Analysis of Medical Student PubMed-Indexed Research Productivity and Factors Associated With Matching at Top-Ranked Ophthalmology Residency Programs

**DOI:** 10.7759/cureus.52824

**Published:** 2024-01-23

**Authors:** Chandruganesh Rasendran, Sanjana Murali, Nithya Kanagasegar, Manasvee Kapadia, Jonathan Lass, Linda Ohsie-Bajor

**Affiliations:** 1 Department of Ophthalmology, University Hospitals Cleveland Medical Center, Cleveland, USA; 2 Department of General Surgery, University of Pennsylvania, Philadelphia, USA

**Keywords:** ophthalmology match, medical student outcomes, ophthalmology residency, sf match, pubmed database

## Abstract

Background

Limited information exists regarding the research productivity of matched ophthalmology applicants given that ophthalmology residencies do not participate in the National Residency Match Program.

Objectives

This study determines the research productivity characteristics of matched ophthalmology applicants and variables associated with matching to higher-tier ophthalmology residency programs.

Methods

Medical school, matched residency program, and applicant-specific PubMed-indexed research productivity (including consideration for first-author publications, relevance to ophthalmology, and acceptance before application submission date) for 2020-2021 matched ophthalmology applicants were collected from publicly available websites. Statistical analysis was conducted using chi-squared tests and t-tests to compare research productivity between groups (gender, medical school tier, and ophthalmology residency program Doximity rank). Multivariate regression was used to analyze research factors associated with matching at the top 20 Doximity-ranked ophthalmology residency programs.

Results

Three hundred ninety-three matched ophthalmology applicants for the 2020-2021 academic year were analyzed with an average of 2.4 ± 3.1 (median: 1 {0-3}) publications, 1.0 ± 2.1 (median: 0 {0-1}) ophthalmic publications, 0.8 ± 1.4 (median: 0 {0-1}) first-author publications, and 0.5 ± 1.1 (median: 0 {0-1}) ophthalmic first-author publications. The applicants who matched at the top 20 Doximity-ranked ophthalmology residency programs were more likely to matriculate from the top 40 medical schools (63% versus 22%, p < 0.001), have more first-author publications at the time of application submission (1.1 ± 1.6 versus 0.7 ± 1.3, p = 0.044), and have more projects resulting in publications after submission (2.0 ± 2.5 versus 1.4 ± 2.3, p = 0.048). In multivariate regression, attendance at a top 40 medical school (adjusted odds ratio {aOR} = 6.07, 95% confidence interval {CI}: 3.56-10.5, p < 0.001) was a significant predictor of matching at a top 20 Doximity ophthalmology residency program, and no variables associated with research productivity were significant predictors.

Conclusions

There has been a consistent increase in research productivity among matched ophthalmology applicants. However, in multivariate analysis, the medical school tier was the only significant variable for matching at top-tier programs. More nuanced studies regarding the effect of research productivity on ophthalmology applicants are needed.

## Introduction

Ophthalmology is consistently considered to be one of the most competitive specialties for the residency match with a rising number of applicants each year. Studies have analyzed the importance of the United States Medical Licensing Examination (USMLE) Step 1 board scores, clinical grades, and Alpha Omega Alpha (AOA) status for matching into ophthalmology [1-3]. The average USMLE Step 1 board scores for accepted applicants have consistently been increasing every year [4], and while involvement in research is regarded as an important metric for an application, there is little information regarding the depth of productivity [2,3,5]. Ophthalmology does not participate in the National Residency Match Program, and consequently, there is no Charting Outcomes in the Match data for the applicants to compare their research productivity and competitiveness to those of past years. The American Academy of Ophthalmology has stated that there is a need for more specific data on the value of research and publications and the importance of devoting elective time to research [6]. Additionally, with the USMLE Step 1 board examination transitioning toward becoming pass/fail in 2022, research output may be an even more important metric for future applicants. Residency programs may also favor applicants with a history of strong research output given the Residency Review Committee’s requirements for ophthalmology residency programs to include a research component within the curriculum, specifically as part of Practice-Based Learning and Improvement competency for programs [7]. Programs may risk penalties such as citation or the loss of accreditation if there is inadequate exposure to research.

Similar analyses on applicant research productivity have recently been completed for other competitive specialties including plastic surgery, otolaryngology, orthopedic surgery, neurosurgery, and urology [8-13]. However, the most recent study on this topic for ophthalmology utilized applicant data from 2014. Given the increasingly competitive nature of the ophthalmology match, there is a need for updated research metrics [14]. The aim of the present study is to quantify the PubMed-indexed research productivity of medical students who matched ophthalmology in the 2020-2021 cycle and to analyze factors associated with matching at top-tier ophthalmology residency programs as determined by Doximity.

## Materials and methods

A widely used and publicly accessible Google (Google, Inc., Mountain View, CA) spreadsheet utilized by ophthalmology applicants during the 2020-2021 interview cycle was used to obtain information regarding the applicants’ medical schools and matched programs. Although this spreadsheet was created and maintained by users, we chose to reference it as our primary source of applicant biographic data, as most residency programs have not yet updated their websites to reflect the newest class of interns and do not uniformly list residents. A Python (Python Software Foundation, Wilmington, DE) programming script was developed to extract information about each applicant’s PubMed-indexed publications, and the results were verified and supplemented manually using PubMed, LinkedIn, ResearchGate, and Google Scholar. Gender API (Passau, Germany), a widely utilized database platform that determines gender by first name, was used to ascertain applicants’ gender [15], and ophthalmology-related keywords were used to identify ophthalmology-related publications by either journal or publication title. Medical schools were stratified by their status as a top 40 US News Medical School: Research (Washington, DC) [16], and the top 20 ophthalmology programs as determined by Doximity (San Francisco, CA) were considered top-tier [17]. These methods of stratifying applicants by medical school and residency programs by Doximity rankings have been utilized in similar literature for other surgical subspecialties [8,10,12].

For each applicant, the number of PubMed-indexed publications, ophthalmology-related publications, first-author publications, impact factors of each publication, gender, the rank of medical school (top 40 versus not), and the rank of the ophthalmology residency program (top 20 on Doximity versus not) were recorded and analyzed. Publications were stratified according to whether they were indexed on PubMed before or after September 15, 2020: the date applications were made available to residency programs. Publications that were published after September 15, 2020, were considered to be active research projects by the applicants at the time of application submission. Statistical analysis was conducted using the R programming language (R Foundation for Statistical Computing, Vienna, Austria) using techniques such as chi-squared tests and t-tests to compare research productivity between groups, and multivariate regression was used to analyze factors associated with matching at the top 20 ophthalmology residency programs [18]. An initial univariate regression model was used to determine which variables to utilize for the multivariate regression model due to the high degree of multicollinearity between the variables pertaining to research productivity such as number of publications, ophthalmology-related publications, first-author publications, and first-author ophthalmology publications and the corresponding variables pertaining to projects that were not yet accepted for publication at the time of submission.

A p-value of <0.05 was considered to be statistically significant. The data and programming scripts used for analysis can be found at github.com/cxr244/OphthoAvgPublications.

## Results

A total of 393 ophthalmology applicants for the 2020-2021 academic year were analyzed or roughly 80% of all matched applicants for that year. Sixty percent of the applicants were male. The average applicant analyzed had 2.4 ± 3.1 (median: 1 {0-3}) publications overall with 1.0 ± 2.1 (median: 0 {0-1}) publications pertaining to ophthalmology (Table 1). The applicants had an average of 0.8 ± 1.4 (median: 0 {0-1}) first-author publications and 0.5 ± 1.1 (median: 0 {0-1}) ophthalmology-related first-author publications. The average impact factor of these publications was 2.8 ± 4.5, and the highest impact factor for all these publications was 5.0 ± 9.0. The average applicant had 1.5 ± 2.4 projects that resulted in publications after applications were submitted of which 1.1 ± 1.9 pertained to ophthalmology. Seventy-nine percent of all the applicants participated in any research scholar activity resulting in an eventual publication. There was no difference in research output based on gender.

**Table 1:** Research Productivity of Ophthalmology Residency Applicants During the 2020-2021 Application Cycle Both in Aggregate and Stratified by Gender Research productivity was assessed for the applicants both at the time of submission (September 15, 2020) and as of June 15, 2021, to assess for ongoing research projects at the time of submission. P-values indicate a difference in the distribution of values between female and male applicants. All values are presented as either n (%) or mean ± SD. Pearson’s chi-squared test and Welch’s two-sample t-tests were used for statistical comparisons

Characteristic	N = 393	Female, N = 158 (40%)	Male, N = 235 (60%)	P-value
Top 40 US News Medical School: Research	115 (30%)	52 (34%)	63 (28%)	0.2
Top 20 Doximity-Ranked Program	80 (20%)	29 (18%)	51 (22%)	0.4
Active Research	311 (79%)	126 (80%)	185 (79%)	0.8
Publications Before Submission				
Publications	2.4 ± 3.1	2.2 ± 2.9	2.5 ± 3.2	0.3
Ophthalmology Publications	1.0 ± 2.1	1.1 ± 2.4	1.0 ± 2.0	0.9
First Authors	0.8 ± 1.4	0.7 ± 1.1	0.8 ± 1.5	0.4
First-Author Ophthalmology Publications	0.5 ± 1.1	0.4 ± 0.9	0.5 ± 1.1	0.6
Highest Impact Factor	5.0 ± 9.0	4.1 ± 7.4	5.6 ± 9.9	0.077
Average Impact Factor	2.8 ± 4.5	2.5 ± 4.1	3.0 ± 4.7	0.2
Ongoing Projects				
Projects	1.5 ± 2.4	1.7 ± 3.0	1.4 ± 1.9	0.2
Ophthalmology Projects	1.1 ± 1.9	1.2 ± 2.3	1.0 ± 1.6	0.3
First-Author Projects	0.7 ± 1.3	0.8 ± 1.3	0.7 ± 1.2	0.3
First-Author Ophthalmology Projects	0.5 ± 1.1	0.6 ± 1.2	0.5 ± 1.0	0.3
Highest Impact Factor of All Projects	6.3 ± 10.0	5.5 ± 9.2	6.7 ± 10.5	0.2
Average Impact Factor of All Projects	3.1 ± 3.8	2.8 ± 3.1	3.3 ± 4.2	0.2

The applicants from the top 40 medical schools when compared to the applicants who did not attend these medical schools had no differences in research output (p > 0.05 for all) but were more likely to match at the top 20 Doximity ophthalmology residency programs (43% versus 11%, p < 0.001) (Table 2). The applicants who matched at the top 20 ophthalmology residency programs were more likely than the applicants who matched at other ophthalmology residency programs to matriculate from the top 40 medical schools (63% versus 22%, p < 0.001), have more first-author publications at the time of application submission (1.1 ± 1.6 versus 0.7 ± 1.3, p = 0.044), and have more projects that resulted in publications after submission (2.0 ± 2.5 versus 1.4 ± 2.3, p = 0.048). In univariate regression, attending a top 40 US News Medical School (adjusted odds ratio {aOR} = 1.37, 95% confidence interval {CI}: 1.26-1.49, p < 0.001), first-author publications at the time of submission (aOR = 1.03, 95% CI: 1.01-1.07, p = 0.022), projects that were not yet accepted by the submission date (aOR = 1.02, 95% CI: 1.00-1.03, p = 0.036), and ophthalmology projects that were not yet accepted by the submission date (aOR = 1.02, 95% CI: 1.00-1.04, p = 0.033) were significant predictors of matching at a top 20 Doximity ophthalmology residency program (Table 3). Active projects and ophthalmology projects that were not yet accepted by the submission date are highly collinear with each other. Therefore, a multivariate regression model was generated with attendance at a top 40 medical school, first-author publications at the time of submission, and active unaccepted projects as the independent variables. Of these variables, only attendance at a top 40 medical school (aOR = 6.07, 95% CI: 3.56-10.5, p < 0.001) was a significant predictor of matching at a top 20 Doximity ophthalmology residency program (Table 4).

**Table 2:** Research Productivity of Ophthalmology Residency Applicants During the 2020-2021 Application Cycle Stratified by Attending a Top 40 US News Medical School or Matching at a Top 20 Doximity Residency Program Research productivity was assessed for the applicants both at the time of submission (September 15, 2020) and as of June 15, 2021, to assess for ongoing research projects at the time of submission. P-values indicate a difference in the distribution of values between the respective sets of stratified applicants. All values are presented as either n (%) or mean ± SD. Pearson’s chi-squared test and Welch’s two-sample t-tests were used for statistical comparisons

	Top 40 Medical School			Top 20 Doximity Program		
Characteristic	No, N = 264	Yes, N = 115	P-value	No, N = 313	Yes, N = 80	P-value
Gender			0.2			0.4
Female	102 (39%)	52 (45%)		129 (41%)	29 (36%)	
Male	162 (61%)	63 (55%)		184 (59%)	51 (64%)	
Top 40 US News Medical School: Research				66 (22%)	49 (63%)	<0.001
Top 20 Doximity-Ranked Program	29 (11%)	49 (43%)	<0.001			
Active Research	208 (79%)	94 (82%)	0.5	244 (78%)	67 (84%)	0.3
Publications Before Submission						
Publications	2.3 ± 3.0	2.6 ± 3.4	0.3	2.3 ± 3.1	2.7 ± 3.1	0.2
Ophthalmology Publications	1.1 ± 2.2	1.0 ± 2.1	0.9	1.0 ± 2.1	1.1 ± 2.1	0.7
First-Author Publications	0.8 ± 1.3	0.9 ± 1.4	0.5	0.7 ± 1.3	1.1 ± 1.6	0.044
First-Author Ophthalmology Publications	0.5 ± 1.0	0.5 ± 1.1	0.9	0.4 ± 1.0	0.6 ± 1.3	0.3
Highest Impact Factor	4.8 ± 8.5	5.5 ± 9.8	0.5	4.7 ± 8.5	6.1 ± 11.0	0.3
Average Impact Factor	2.7 ± 4.4	3.1 ± 4.7	0.5	2.7 ± 4.5	3.0 ± 4.4	0.6
Ongoing Projects						
Projects	1.4 ± 2.3	1.6 ± 2.2	0.5	1.4 ± 2.3	2.0 ± 2.5	0.048
Ophthalmology Projects	1.1 ± 2.0	1.2 ± 1.9	0.6	1.0 ± 1.8	1.5 ± 2.2	0.056
First-Author Projects	0.7 ± 1.2	0.7 ± 1.2	0.5	0.7 ± 1.2	0.9 ± 1.5	0.13
First-Author Ophthalmology Projects	0.5 ± 1.1	0.6 ± 1.1	0.6	0.5 ± 1.0	0.7 ± 1.3	0.14
Highest Impact Factor of All Projects	5.9 ± 9.4	7.1 ± 11.0	0.3	6.0 ± 9.8	7.3 ± 10.7	0.3
Average Impact Factor of All Projects	2.9 ± 3.3	3.5 ± 4.5	0.15	3.0 ± 3.8	3.4 ± 3.7	0.4

**Table 3:** Univariate Regression Model for the Predictors of an Applicant Matching at a Top 20 Doximity Ophthalmology Residency Program Research productivity was assessed for the applicants both at the time of submission (September 15, 2020) and as of June 15, 2021, to assess for ongoing research projects at the time of submission. P-values indicate the significance of the respective variables and values reported in a 95% confidence interval (CI).

Characteristic	N	Odds Ratio	95% CI	P-value
Gender	393			
Female		-	-	
Male		1.03	0.95-1.12	0.4
Top 40 US News Medical School: Research	379	1.37	1.26-1.49	<0.001
Active Research	393	1.06	0.96-1.17	0.3
Publications Before Submission				
Publications	393	1.01	0.99-1.02	0.2
Ophthalmology Publications	393	1.00	0.98-1.02	0.7
First-Author Publications	393	1.03	1.01-1.07	0.022
First-Author Ophthalmology Publications	393	1.03	0.99-1.06	0.2
Highest Impact Factor	393	1.00	1.00-1.01	0.2
Average Impact Factor	393	1.00	0.99-1.01	0.6
Ongoing Projects				
Projects	393	1.02	1.00-1.03	0.036
Ophthalmology Projects	393	1.02	1.00-1.04	0.033
First-Author Projects	393	1.03	1.00-1.06	0.090
First-Author Ophthalmology Projects	393	1.03	0.99-1.07	0.093
Highest Impact Factor of All Projects	393	1.00	1.00-1.01	0.3
Average Impact Factor of All Projects	393	1.00	0.99-1.02	0.4

**Table 4:** Multivariate Regression Model Utilizing Variables That Were Significant in Univariate Regression Model for Matching at a Top 20 Doximity Ophthalmology Residency Program Due to the high degree of collinearity between the number of projects and ophthalmology-related projects, ophthalmology-related projects were excluded in the multivariate analysis. Variables are presented as odds ratio or confidence intervals (CI)

Characteristic	Odds Ratio	95% CI	P-value
Top 40 US News Medical School: Research	6.07	3.56-10.5	<0.001
First-Author Publications	1.17	0.97-1.40	0.10
Projects in Progress at Submission	1.09	0.98-1.21	0.11

There were 691 total ophthalmology-related publications by the applicants (Table 5). The most common ophthalmology journals that published medical student publications were the American Journal of Ophthalmology (55), American Journal of Ophthalmology Case Reports (47), Ophthalmology Retina (44), and Clinical Ophthalmology (35).

**Table 5:** Ten Ophthalmology Journals Associated With the Most Publications

Journal	Publications
American Journal of Ophthalmology	55
American Journal of Ophthalmology Case Reports	47
Ophthalmology Retina	44
Clinical Ophthalmology (Auckland, New Zealand)	35
Ophthalmology	34
Ophthalmic Plastic and Reconstructive Surgery	33
Investigative Ophthalmology & Visual Science	32
JAMA Ophthalmology	31
Retina (Philadelphia, PA)	27
Cornea	27
Total for All Ophthalmology Journals	691

## Discussion

With the increasing competitiveness of the ophthalmology residency match and the paucity of objective metrics by which to compare the applicants, there is a need for increased clarity regarding the research productivity of the most recently matched applicants. In our analysis of the PubMed-indexed publications of the 80% of matched ophthalmology applicants in the 2020-2021 academic year, the applicants had roughly 2.4 publications (likely right-skewed by certain applicants with a large volume of publications signified by a median of 1.0 publication for the accepted class) of which 1.0 pertain to ophthalmology and 0.8 are first-author publications. The applicants who matched at the top 20 Doximity ophthalmology residency programs were more likely to attend a top 40 US News Medical School and have more first-author publications and active projects pertaining to ophthalmology that later resulted in publications; however, in multivariate regression analysis including the aforementioned variables, only attending a top 40 medical school increased the likelihood of an applicant matching at a top-tier ophthalmology residency program, suggesting that research productivity as an independent variable may not be significant in matching to a top-tier ophthalmology residency program especially since there were no differences in research on outpatient based on the applicants’ tier of medical school.

Bargoud et al.’s study, which was the only similar study of ophthalmology applicants, utilized ophthalmology applicant data from 2014 and reported an average of 1.23 publications for 340 of 464 total ophthalmology applicants (73% of the total applicants from 2014), and per our analysis, the average number of publications has increased for the 2020-2021 cohort [14]. For the neurosurgery applicants, a similar trend was noted with an increase in the number of publications based on applicant year (2.6 for neurosurgery interns in 2014, 3.8 in 2016, and 5.5 in 2018), suggesting an applicant “arms race” toward increased research productivity [9]. This trend may only be exacerbated by USMLE Step 1 becoming pass/fail and the lack of metrics by which to compare the applicants.

Attending a top 40 medical school was associated with an increased likelihood of matching at a top-tier residency program, as ranked by Doximity, and has previously been reported as an important predictor for matching into ophthalmology residency [1]. The results of the present study suggest that attending a top 40 US News Medical School is an important predictor of matching at a top 20 Doximity ophthalmology residency program. Interestingly, research productivity was not associated with a higher likelihood of matching at a top-tier ophthalmology residency program in multivariate analysis. Medical students at top medical schools may interact with faculty that are more well-known to top residency programs and consequently may have an impactful letter of recommendation in addition to more research funding for research projects [5,19].

A limitation of this study is the lack of access to San Francisco match data, but self-reported data from over 80% of the incoming class was analyzed in this study. Research that was not PubMed-indexed was not included in this study, and thus, the impact of abstracts, posters, oral presentations, patents, and publications in journals that were not indexed on PubMed was not analyzed. We chose to narrow our focus to PubMed-indexed publications to remain as objective as possible since there is no centralized source of data reporting applicant abstracts, posters, and oral presentations. There is a need for further research to study the relationship between involvement in research and the quality of letters of recommendation, as well as further research on the importance of abstracts and oral and poster presentations when evaluating applicant research productivity. Doximity ranking was utilized as a surrogate for top-tier ophthalmology residency programs. However, Doximity ranks residency programs based on resident/alumni satisfaction surveys and reputation data where board-certified physicians can nominate residency programs, which may not necessarily be the same as research productivity or training quality of residency programs serving as a limitation of this study [20]. Another limitation of this study is the lack of access to USMLE Step 1 examination scores, letters of recommendation, and clinical grades for applicants.

## Conclusions

There has been a consistent increase in research productivity among ophthalmology applicants. In multivariable analysis, overall research productivity was not a predictive variable, and there needs to be further research regarding the role of first-author publications versus ophthalmology publications and other aspects of research productivity in matching at the top 20 Doximity ophthalmology residency programs. This analysis enables future applicants and program directors to better assess the role of research in the application process while providing avenues for future studies in this arena.
